# Evaluation of percutaneous renal biopsy complications based on outcomes and indicators of the Nursing Outcomes Classification*

**DOI:** 10.1590/1518-8345.3759.3415

**Published:** 2021-07-02

**Authors:** Magáli Costa Oliveira, Fernanda Da Silva Flores, Franciele Moreira Barbosa, Cinthia Dalasta Caetano Fujii, Eneida Rejane Rabelo-Silva, Amália de Fátima Lucena

**Affiliations:** 1 Universidade Federal do Rio Grande do Sul, Escola de Enfermagem, Porto Alegre, RS, Brazil.; 2 Hospital de Clínicas de Porto Alegre, Serviço de Nefrologia, Porto Alegre, RS, Brazil.

**Keywords:** Outcome Assessment, Health Care, Nursing Process, Classification, Biopsy, Complications, Nephrology Nursing, Evaluación de Resultado em la Atención de Salud, Proceso de Enfermería, Clasificación, Biopsia, Complicaciones, Enfermería em Nefrología, Avaliação de Resultados em Cuidados de Saúde, Processo de Enfermagem, Classificação, Biópsia, Complicações, Enfermagem em Nefrologia

## Abstract

**Objective:**

to evaluate the complications of percutaneous renal biopsy based on outcomes and clinical indicators of the Nursing Outcomes Classification.

**Method:**

a prospective longitudinal study. The sample consisted of 13 patients submitted to percutaneous renal biopsy, with 65 evaluations. The patients were evaluated in five moments in the 24 hours after the procedure, using an instrument developed by the researchers based on five outcomes (Blood coagulation, Circulation status, Blood loss severity, Pain level, Comfort status: Physical) and 11 indicators. The Generalized Estimation Equation Test was used to compare the scores of the indicators. The project was approved by the institutional ethics committee.

**Results:**

in the 65 evaluations, a statistically significant difference was identified in the reduction of the scores of the following nursing outcomes: Blood coagulation, “hematuria” indicator; Circulation status, in the “systolic blood pressure and diastolic blood pressure” indicators and Comfort status: physical, in the “physical well-being” indicator.

**Conclusion:**

the evaluated patients did not show major complications. The clinical indicators signaled changes in circulation status, with reduced blood pressure, as well as in blood clotting observed by hematuria, but without hemodynamic instability. The comfort status was affected by the rest time after the procedure.

## Abstract (es)

**Objetivo:**

evaluar las complicaciones de la biopsia renal percutánea con base en los resultados e indicadores clínicos de la *Nursing Outcomes Classification*.

**Método:**

estudio longitudinal prospectivo. La muestra fue de 13 pacientes a los que se les realizó biopsia renal percutánea, con 65 evaluaciones. Los pacientes fueron evaluados en cinco momentos en las 24 horas posteriores al procedimiento, utilizando un instrumento desarrollado por los investigadores con base en cinco resultados (Coagulación sanguínea, Estado circulatorio, Severidad de la pérdida de sangre, Nivel de dolor, Estado de comodidad: física) y 11 indicadores. Se utilizó la Prueba de Ecuación de Estimación Generalizada para comparar los puntajes de los indicadores. El proyecto fue aprobado por el comité ético institucional.

**Resultados:**

en las 65 evaluaciones, se identificó una diferencia estadísticamente significativa en la reducción de los puntajes de los resultados de enfermería Coagulación sanguínea, indicador “hematuria”; Estado circulatorio, en los indicadores “presión arterial sistólica y presión arterial diastólica” y en el Estado de comodidad: física, en el indicador de “bienestar físico”.

**Conclusión:**

los pacientes evaluados no presentaron mayores complicaciones. Los indicadores clínicos apuntaban a cambios en el estado circulatorio, con reducción presión arterial, así como en la coagulación sanguínea verificada por hematuria, pero sin inestabilidad hemodinámica. El estado de comodidad se vio afectado por el tiempo de descanso posterior al procedimiento.

## Resumo

**Objetivo:**

avaliar as complicações da biópsia renal percutânea com base nos resultados e indicadores clínicos da *Nursing Outcomes Classification.*

**Método:**

estudo longitudinal prospectivo. A amostra foi de 13 pacientes submetidos à biópsia renal percutânea, com 65 avaliações. Os pacientes foram avaliados em cinco momentos nas 24 horas após o procedimento, por meio de um instrumento desenvolvido pelos pesquisadores com base em cinco resultados (Coagulação sanguínea, Estado circulatório, Gravidade da perda de sangue, Nível de dor, Estado de conforto: físico) e 11 indicadores. Utilizou-se o Teste de Equações de Estimação Generalizadas para comparação entre os escores dos indicadores. O projeto foi aprovado pelo comitê de ética institucional.

**Resultados:**

nas 65 avaliações foi identificada diferença estatisticamente significativa na redução dos escores dos resultados de enfermagem Coagulação sanguínea, indicador “hematúria”; Estado circulatório, nos indicadores “pressão arterial sistólica e pressão arterial diastólica” e no Estado de conforto: físico, no indicador “bem-estar físico”.

**Conclusão:**

os pacientes avaliados não apresentaram complicações maiores. Os indicadores clínicos apontaram alterações no estado circulatório, com redução da pressão arterial, bem como na coagulação sanguínea constatada pela hematúria, porém sem instabilidade hemodinâmica. O estado de conforto foi afetado pelo tempo de repouso após o procedimento.

## Introduction

Percutaneous renal biopsy (PRB) is an important procedure for the diagnosis, prognostic evaluation and therapeutic guidance of diverse kidney diseases^1^^-^ 2. Although it is considered a safe procedure, complications may come to occur and, in most cases, are related to the risk of bleeding^3^.

These complications are divided between major and minor. The major ones include macroscopic hematuria and retroperitoneal hematoma requiring blood transfusions, surgical interventions or invasive procedures. Minor complications consist of transient macroscopic or microscopic hematuria, without the need for transfusion or other interventions, nephrectomy and bladder obstruction^1^^-^ 2. Arteriovenous fistulas, infection, puncture or damage of other organs may also come to occur and death, very rarely^1^^-^ 3.

The clinical evaluation of the patient submitted to PRB by the nurse includes the monitoring of signs and symptoms, aiming to identify early potential complications to avoid and/or decrease their incidence, as well as facilitate patient recovery. The use of a classification system, based on a standardized language, allows nurses to diagnose, plan, intervene and evaluate the results obtained. 

The Nursing Outcomes Classification (NOC)^4^ is one of these classification systems, which presents the standardization of nursing outcomes and indicators and has been explored in different content validation studies, which have demonstrated its applicability and benefits in the accuracy of nursing assessments^5^^-^ 8. However, it is indispensable that clinical validation studies also be developed in different care-related settings, to support and incorporate the use of this classification in the professional practice. 

In this sense, a number of studies also pointed out that the NOC^4^ made it possible to demonstrate the clinical evolution of the patients in different care contexts^9^^-^ 11. It is known that the precise assessment of the patient’s health status, as well as the effectiveness of the interventions performed, favors the construction of evidence and, consequently, can influence the reduction of the patient’s hospitalization time and the resulting hospital costs^10^. 

A systematic review on standardized language identified 312 articles, most of them on the NOC with a focus on the reliability or validity of its terms and nurses’ perception of their potential to be used in the practice. However, only six studies used this classification in the clinical nursing practice^12^. In view of the above, together with the fact that although there are studies on the applicability of the NOC^4^ there are still no researches on the use of this classification as an alternative to be used in the more accurate and safe evaluation of patients submitted to PRB, allowing a monitoring capable of avoiding and/or minimizing the complications resulting from this procedure, the present study was conducted. 

Thus, the study was developed in a real clinical setting and aimed to evaluate the complications of PRB based on outcomes and clinical indicators of the NOC.

## Method

This is a longitudinal study with prospective data collection^13^, conducted in a large public university hospital, located in southern Brazil. The fields of research were the Outpatient Surgical Center, the Radiology Unit, the Hemodialysis Unit and the Hospitalization Units. 

Patients were recruited for the study occurred according to the marking schedule biopsies, informed daily to the researchers, by the research field nurses. Patients of both genders, aged 18 years old or over and submitted to PRB, were included. Those who were submitted to more than four punctures during the procedure and/or use of a larger needle (14G) were excluded, due to the fact that these patients have medical indication to continue resting in bed rest for 24 hours after the procedure. Bedridden patients or who were unable to walk were also excluded, because the evaluation of the “Comfort status: Physical” nursing outcome is related to the patient leaving the bed. 

The sample was calculated using the WINPEPI program, version 11.43. The same was estimated for the outcome of the main complications of PRB, knowing that bleeding is the most frequent of them. The NOC^4^ was also considered in relation to the selected clinical indicators, considering a difference of 1 point in the SCORE of the NOC^4^ in the evaluations, with 90% power and alpha error of 5%, standard deviation between scores of 1 and correlation stipulated between the first and last evaluation of 0.5, based on previous studies^9^^-^ 11 ^,^ 14. Thus, the stipulated sample was 13 patients with 65 evaluations, adding up 20% of losses.

Data collection took place from February to May 2018. The data collection instrument was built by the researchers and consisted of sociodemographic and clinical characteristics of the patients, description of the biopsies performed and NOC nursing outcomes and indicators^4^.

The selection of indicators and outcomes was carried out by the researchers based on the literature^1^^-^ 3, which describes the potential complications after PRB and according to the consensus of 12 nurses specialized in the field of Nephrology with clinical expertise in caring for these patients. The specialists had a median training time of 18 years and 6.5 years of professional experience, which corroborates their clinical experience about the risks and needs of care for patients undergoing PRB. Some of them also presented an expressive number of publications, especially in surveys aimed at nursing taxonomies, enabling them to give a proper opinion on the topic under study.

For each indicator of the NOC^4^ selected, conceptual and operational definitions were developed, also based on the literature, in order to reduce the subjectivity of the application of their scores in the assessment of patients. Thus, this part of the instrument contained five outcomes and 11 indicators from the NOC^4^ (Figure 1), with their respective 5-point Likert type scales, whose lowest score represents the worst state and the highest score the best state. The application of the instrument was carried out at the patient’s bedside, in a real clinical setting, by one of the researchers, who is a nurse, with the help of scientific initiation scholarship fellows and nursing graduates, duly trained for this.

**Figure 1:** Nursing outcomes and indicators from the Nursing Outcomes Classification selected for the evaluation of the patient undergoing percutaneous renal biopsy. Southern Brazil, 2018

Nursing Outcomes (numeric code)	Clinical Indicators (numeric code)
Blood coagulation (0409)	Bleeding (040902) Bruising (040903) Hematuria (040918)
Circulation status (0401)	Systolic blood pressure (040101) Diastolic blood pressure (040102)
Blood loss severity (0413)	Abdominal distension (041306) Skin and mucous membrane pallor (041313)
Pain level (2102)	Reported pain (210201) Facial expressions of pain (210206)
Comfort status: physical (2010)	Physical well-being (201002) Comfortable position (201004)

Figure 1 - Nursing outcomes and indicators from the Nursing Outcomes Classification selected for the evaluation of the patient undergoing percutaneous renal biopsy. Southern Brazil, 2018

All the patients were evaluated at five different times by the researchers in a total period of 24 hours: immediately before the procedure (A0), to know the baseline status of the patient; immediately after PRB (A1); at the eighth hour after the procedure (A2); at the 12th hour (A3) and at the 24th hour after PRB (A4). The interval between evaluations was determined based on studies on bed rest time after PRB, which indicate that complications are more frequent in the first hours and up to 24 hours after the procedure^1^^-^ 2.

The data were organized in *Excel for Windows* and analyzed in the *Statistical Package for the Social Sciences*, version 21.0. The continuous variables were described as mean and standard deviation or as median and interquartile range, according to data distribution. The categorical variables were described by means of absolute and relative frequencies. To compare the mean scores of the nursing indicators over time, the Generalized Estimation Equations model^15^^)^ was applied with Bonferroni adjustment to locate the differences between the means. The level of significance adopted was 5% (p<0.05).

The project was submitted to Brazil Platform and approved by the institution’s Research Ethics Committee (Protocol No. 170430). The specialists who participated in the selection stage of the clinical indicators used in the study consented to their participation by submitting their answers using an online form. The patients who took part in the study signed two copies of the Free and Informed Consent Form.

## Results

Of the 13 patients submitted to BRP, the following were identified: seven (54%) male patients were identified and seven white-skinned (54%), with a mean age of 46.6 (± 12.3) years old. Eight (61.5%) were hypertensive, four (30.8%) diabetic and six (46.2%) kidney transplant recipients. Four (30.8%) of the patients had already performed the procedure on another occasion (Table 1).

**Table 1:** Sociodemographic and clinical characteristics of the patients undergoing percutaneous renal biopsy (n=13). Southern Brazil, 2018

Variables		n=13 (%)
Age (years old)*		46.6 ± 12.3
Gender		
	Male	7 (54)
Skin color		
	White	7 (54)
	Black	3 (23.1)
	Brown	3 (23.1)
BMI^†^ (kg/m^2^)*		26.1 ± 4.0
Comorbidities		
	Hypertension	8 (61.5)
	Renal transplant	6 (46.2)
	Diabetes	4 (30.8)
Previous renal biopsy		4 (30.8)

* Mean ± standard deviation;

† BMI = Body Mass Index

The performance of 53.8% of the procedures occurred in the Outpatient Surgical Center, with patients coming from their home and who remain for 24 hours under observation after the interventions. The predominance of procedures performed at this site is of native kidneys, due to the need to investigate the etiology of renal function loss. Regarding the needles gauge used, 12 (92.3%) were of 16G gauge, both in native kidney and graft (Table 2).

**Table 2:** Characterization of percutaneous renal biopsies of patients submitted to this intervention (n=13). Southern Brazil, 2018

Variables		n=13 (%)
Type of biopsied kidney		
	Native	7 (53.8)
	Graft	6 (46.2)
Performance unit		
	Outpatient Surgical Center	7 (53.8)
	Hemodialysis	5 (38.5)
	Radiology	1 (7.7)
Number of punctures*		2.5 ± 0.8
Needle gauge		
	16G	12 (92.3)
	18G	1 (7.7)

* Mean ± standard deviation

Table 3 displays the mean scores of the outcomes and their indicators of the patients evaluated according to the 5-point Likert type scale, as per the NOC^4^.

**Table 3:** Mean scores of the nursing outcomes and their indicators in evaluating the patients submitted to percutaneous renal biopsy (n=13). Southern Brazil, 2018

	Outcomes/ Indicators NOC	A0 Before	A1 Soon afterwards	A2 8 h after	A3 12 h after	A4 24 h after	p
		Mean±SD	Mean±SD	Mean±SD	Mean±SD	Mean±SD	
Blood coagulation		4.6 ± 0.4^b^	-	4.4 ± 0.4a	4.5 ± 0.2ab	4.7 ± 0.5b	0.018
	Bleeding	5.0 ± 0.0	4.8 ± 0.4	5.0 ± 0.0	5.0 ± 0.0	5.0 ± 0.0	*
	Bruising	5.0 ± 0.0	5.0 ± 0.0	5.0 ± 0.0	5.0 ± 0.0	5.0 ± 0.0	*
	Hematuria	4.0 ± 1.2^b^	-	3.0 ± 1.1^a^	3.5 ± 0.6^ab^	4.0 ± 1.4^b^	0.018^‡^
Circulation status		5.0 ± 0.0^c^	4.7 ± 0.8^bc^	3.7 ± 1.3^a^	3.8 ± 1.3^ab^	3.7 ± 1.5^ab^	<0.001
	Systolic blood	5.0 ± 0.0^c^	4.7 ± 0.9^bc^	3.5 ± 1.6^a^	3.9 ± 1.5^ab^	3.9 ± 1.6^abc^	0.001
	Diastolic blood pressure	5.0 ± 0.0^c^	4.7 ± 0.8^bc^	3.8 ± 1.2^a^	3.8 ± 1.4^ab^	3.6 ± 1.6^ab^	<0.001^†^
Blood loss severity		4.8 ± 0.3	4.7 ± 0.4	4.7 ± 0.4	4.7 ± 0.4	4.7 ± 0.4	0.272
	Abdominal distension	4.8 ± 0.6	4.8 ± 0.6	4.7 ± 0.6	4.7 ± 0.6	4.7 ± 0.6	0.298
	Skin and mucous membrane pallor	4.8 ± 0.4	4.6 ± 0.7	4.7 ± 0.5	4.8 ± 0.4	4.8 ± 0.4	0.307
Pain level		4.8 ± 0.4	4.8 ± 0.5	4.9 ± 0.1	4.9 ± 0.1	5.0 ± 0.0	0.094
	Reported Pain	4.7 ± 0.8	4.8 ± 0.6	4.9 ± 0.3	4.9 ± 0.3	5.0 ± 0.0	0.107
	Facial expressions of pain	5.0 ± 0.0	4.9 ± 0.4	5.0 ± 0.0	5.0 ± 0.0	5.0 ± 0.0	*
Comfort status: Physical		4.9 ± 0.1^ab^	4.9 ± 0.1^b^	4.6 ± 0.5^a^	4.7 ± 0.4^ab^	4.6 ± 0.5^a^	0.004
	Physical well-being	4.9 ± 0.3^ab^	4.9 ± 0.3^b^	4.6 ± 0.5^a^	4.7 ± 0.5^ab^	4.5 ± 0.7^ab^	0.044
	Comfortable position	5.0 ± 0.0	5.0 ± 0.0	4.6 ± 0.5	4.8 ± 0.4	4.7 ± 0.5	*

* It was not possible to perform the statistical test due to lack of variability; ^a,b,c^Equal letters do not differ by the Bonferroni test at 5% significance;

† Patient in sleep without other complications;

‡ This indicator was applied to 11 patients

The Blood coagulation, Circulation status and Comfort status: physical nursing outcomes were statistically significant. In the “bleeding, bruising, facial expressions of pain and comfortable position” indicators, it was not possible to perform the statistical test due to lack of variability in their scores, which remained high. In the “abdominal distension, skin and mucous membrane pallor and reported pain” indicators, there was no significant difference between the moments evaluated, which also points to the absence of complications after the procedure. There was a significant change in the scores of four clinical indicators: “hematuria, systolic blood pressure, diastolic blood pressure and physical well-being”. 

As for “hematuria”, considering the scores obtained before PRB, it was observed that these were significantly higher than in the eight-hour period after the procedure. However, this indicator was the only one that could not be applied in the 65 evaluations performed, as in some cases the patient did not show diuresis at the time of the evaluation. This situation is justified by the fact that the sample studied is composed by patients with chronic kidney disease, in which change in urinary volume is usually found. Another factor that interferes with urinary volume is due to the patient’s fasting 4 hours before the procedure and 4 hours after, that is, without fluid intake. 

Regarding “systolic blood pressure and diastolic blood pressure”, the scores at the 8th hour and at the 12th hours after PRB were substantially lower than at the pre- and immediately post-procedure. In addition, “diastolic blood pressure” also had a considerably lower score at the 24th hour, when compared to the moment before the intervention. With regard to “physical well-being”, the scores at the 8th hour after PRB were significantly lower than after the procedure, with a slight difference in the 12th and 24th evaluations after, being less than one point on the scale of the NOC^4^.

## Discussion

This is the first study developed in a real clinical setting on the application of the NOC^4^ in patients undergoing PRB. The results showed that the patient undergoing this type of procedure is at risk, with the possibility of complications, but not necessarily with changes. Therefore, it is expected that the indicators will remain with high scores. Monitoring allows the nurse to have enough elements to know the possible complications and to know the risk factors, enabling their prevention.

The stage of validation by expert consensus was essential for the development of the study, since the NOC^4^ has several outcomes and indicators, without determining which ones are most related to each clinical situation. Therefore, the selection was made considering the specificities of the profile of patients submitted to PRB, as in the case of studies carried out in other care practice settings^9^^-^ 11 ^,^ 16. In addition, the consensus enabled the discussion and analysis of issues from different situations to reach an agreement between the expert professionals. 

Thus, nursing outcomes and clinical indicators were selected for which conceptual and operational definitions were built, according to the magnitude of the 5-point Likert scale of the NOC^4^ and which supported their application in a real clinical setting. The construction of these definitions is very important in order to have an assessment without subjectivity and also to standardize the application in the clinical practice^9^^-^ 11. The clinical indicators selected are in accordance with the signs and symptoms already described in the literature as possible complications of PRB^3^^,^ 17 ^-^ 20. They also include important aspects for their supervision and monitoring, demonstrating that the NOC^4^ has indicators that favor the evaluation of these patients and that can qualify the assistance provided. 

Regarding the characteristics of the patients, the study sample consisted, predominantly, by those with chronic diseases with chronic diseases, with half of the sample being kidney transplanted, which is corroborated by the literature^18^^,^ 21 ^-^ 22. 

Biopsies were performed on both native kidneys and kidney grafts, which did not interfere with the occurrence of complications, since all the patients remained well, without complications. Differently from the present study, the literature on rates of complications after PRB points out that those performed in native kidneys are higher compared to allografts, because the latter are easier to access, in addition to allowing easier compression of the site, in bleeding cases^18^^,^ 22.

Also regarding the procedure, all the biopsies were performed with a smaller gauge needle, with 12 (92.3%) with a 16G gauge needle and one (7.7%) with 18G gauge, which is known to reduce the risk of bleeding, when compared to the use of larger gauge needles^2^^-^ 3 ^,^ 19 ^,^ 23. Corroborating this finding, another study compared the use of 14G and 16G gauge needles, indicating that the 16G result in fewer post-biopsy hematomas and have a diagnostic yield equivalent to 14G for PRB^24^. However, the ideal practice is to puncture by using biopsy needles with spring, under direct radiological guidance (by ultrasound), as they have a more favorable risk profile as compared to the other devices^19^.

Regarding the evaluation on the complications of the procedure, with a NOC-based instrument^4^, the scores obtained indicated effective supervision and the absence of major complications. Systematic monitoring with outcomes and clinical indicators focused on the assessment of signs and symptoms, especially in relation to the risk of bleeding, allowed showing that the biopsied patients are being well-assisted, according to the care protocol existing in the study field institution, which includes the measurement of vital signs at regular intervals in the first four hours and maintenance of rest, among others.

In the Blood coagulation nursing outcome, the “bleeding” clinical indicator remained with its scores practically unchanged over time, with a slight decrease occurring shortly after the procedure. As for the “bruising” indicator, no patient presented this complication, that is, the scores remained at their maximum value in all assessments. It is worth noting that the scores measured during the evaluations can vary from positive (increase in the scale score), negative (decrease) or, even, there may be no change. For certain situations in which the patient does not have conditions for improvement, the goal ends up being to maintain the clinical status at a certain magnitude of the outcome^4^.

In addition to nursing care and monitoring of potential biopsy complications, it is relevant to note that the technique used in performing the procedure, as well as the prediction of risk factors, are aspects that also minimize the chances of the patient presenting complications^2^^-^ 3 ^,^ 22 ^,^ 25. According to the literature, the advancement of technology with the use of automatic devices and of smaller gauge needles, as well as the use of real-time ultrasound to perform the puncture, greatly reduced the complications of biopsy^2^^,^ 19 ^,^ 26 ^-^ 28. It is noteworthy, therefore, that in the study field hospital, PRB are performed according to this technique, which increases patient safety and decreases the incidence of complications. 

Regarding the “hematuria” indicator, the scores before the biopsy and at the 24th hour were significantly higher than at the eighth hour. However, it should be noted that this was the only indicator not measured in the total sample of patients (n=13), considering that two patients did not want or could not urinate in any of the five evaluated moments. In addition, at the 8th hour after the procedure, the largest number of evaluations was performed with the tape test to check for microhematuria and inspection in relation to the presence of macrohematuria (eight patients), which justifies the fact that there was a greater difference at this moment. According to the literature^29^, hematuria is common after biopsies and most cases resolve spontaneously. In the patients who participated in the research, hematuria was a minor complication, with no need for additional intervention or treatment.

As for the Circulation status nursing outcome, assessed by the “systolic blood pressure and diastolic blood pressure” indicators, it is highlighted that the values found at the eighth and 12th hours after the procedure were obtained in the early evening and during the night, which can explain the lower blood pressure values when compared to the patient’s baseline (before biopsy) or with the evaluation performed 24 hours after the procedure, which always happened during daytime hours when the patient was awake. It is known that the pressure values are lower during sleep, but without other associated clinical repercussions^30^^-^ 31. Therefore, an indicator should not be evaluated dissociated from a set of other clinical indicators, in order to obtain a reliable evaluation of an outcome^4^. 

A number of studies indicate that blood pressure varies widely during 24 hours and during sleep, in healthy individuals, there is a progressive decrease in their values^30^. Blood pressure varies according to the circadian cycle, presenting nocturnal decline, whose normal value corresponds to a reduction of, at least, 10% in blood pressure during sleep in relation to wakefulness^31^. In the studied population, no patient had any other hemodynamic symptoms associated with a drop in systolic and diastolic blood pressure. Although the measurements took place after the patient awakened at the time of the assessment, the resting state and the characteristics of the environment (silence, lights off or low) may have caused this variation, without blood pressure being associated with any complication of the PRB. 

In the Blood loss severity nursing outcome, the scores for the “abdominal distension and skin and mucous membrane pallor” indicators did not indicate a statistically significant difference in any evaluation during the 24 hours after PRB. This points out to the unchanged status of the patient, that is, the absence of these signs and symptoms after the procedure. This finding does not exclude the importance of measuring these two indicators pointed out by specialist nurses as priorities for patient assessment after PRB, since they are signs and symptoms that can indicate the occurrence of major complications, when associated with other clinical indicators^29^.

Regarding the Pain level outcome, the “reported pain and facial expressions of pain” indicators showed high scores, demonstrating the absence of pain in all the assessments performed over the 24 hours. Thus, it is inferred that the patients were well-assisted by the health team, with the implementation of comfort and analgesia measures, when necessary. 

Pain management by the nurse is fundamental for the patient’s good recovery, since it is a symptom that can trigger psychological and physiological changes that can worsen their health situation. Thus, pain control and relief are essential, with appropriate pharmacological and non-pharmacological interventions for each case^32^, in order to guarantee the well-being of the patients.

As for the Comfort status: Physical nursing outcome, the “physical well-being” indicator at the 8th hour after the procedure had a score significantly lower than in the initial evaluations (p<0.044). Physical well-being refers to the general state of physical comfort and the perception of this well-being by the patient^33^. In the present study, physical well-being was measured through observation and questioning the patient regarding the following characteristics: good physical mobility, feeling comfortable, normal breathing, controlling fatigue and appetite after releasing the diet, among other characteristics observed at the time of the evaluation. 

It became evident that, as the hours passed, there was a change in the patient’s feeling of comfort and physical well-being, possibly associated with the long rest period after the biopsy, which includes movement restriction in the first hours after the procedure, in addition to bed rest for 24 hours. 

The set of outcomes and indicators applied in this study reflects the clinical condition of the patients after PRB, pointing to their care needs^16^. When assessing the patients after PRB, with standardized instruments, the nurse will be able to identify, early and more reliably, the possible complications resulting from this procedure, as well as to prevent them or avoid them from worsening. The study findings point to the selection of the main elements for the evaluation of patients undergoing PRB, through a set of possible outcomes and indicators to be applied, in this clinical setting.

The study presented as a limitation the fact that the evaluations at the eighth and 12th hours always occur at night and/or at dawn, which may have interfered in the evaluation of some indicators, such as the measurement of blood pressure during sleep. It was not possible to modify this logistics because the vast majority of the biopsies, in the field hospital of the study, are performed in the afternoon shift. That is, the observation period of the patient, which begins with the procedure and progresses for 24 hours and ends up only on the following day. Another limitation was the difficulty of measuring the indicator of microscopic or macroscopic hematuria, since not all patients urinated at the five moments evaluated. 

## Conclusion

The outcomes and clinical indicators selected and evaluated in a real care setting are in line with the literature regarding possible complications of PRB, allowing us to conclude that the evaluated patients had no major complications. The clinical indicators signaled changes in circulation status, with reduced blood pressure, as well as in blood clotting observed by hematuria, but without hemodynamic instability. The comfort status was affected by the rest time, after the procedure.

Thus, it is inferred that there has been effective monitoring of the patients after the procedure. The set of evaluated outcomes and indicators points out to the specificity of the Nursing care in this setting of clinical practice and provides subsidies to qualify and enable a more reliable assessment of patients at risk of complications after PRB, in addition to promoting patient safety in real clinical setting. 

Therefore, this study demonstrated the feasibility of applying the NOC in the Nursing practice, which confirms the importance of taxonomies combined with the Nursing Process in the fields of care, teaching and research. The comparison of these findings with future research studies will allow for the refinement of the use of this taxonomy in the clinical setting of patients undergoing PRB.
